# Resolution Enhancement in Surface Plasmon Resonance Sensor Based on Waveguide Coupled Mode by Combining a Bimetallic Approach

**DOI:** 10.3390/s101211390

**Published:** 2010-12-13

**Authors:** Kyeong-Seok Lee, Ju Myeong Son, Dae-Yong Jeong, Taek Sung Lee, Won Mok Kim

**Affiliations:** 1 Electronic Materials Center, Korea Institute of Science and Technology, Seoul 136-791, Korea; E-Mails: tslee@kist.re.kr (T.L.); wmkim@kist.re.kr (W.K.); 2 Department of Materials Science and Engineering, Myongji University, Yongin 443-749, Korea; E-Mails: t7284@naver.com (J.S.); dyjeong@mju.ac.kr (D.J.)

**Keywords:** waveguide coupled surface plasmon resonance, bimetallic approach, high resolution SPR sensor

## Abstract

In this study, we present and demonstrate a new route to a great enhancement in resolution of surface plasmon resonance sensors. Basically, our approach combines a waveguide coupled plasmonic mode and a kind of Au/Ag bimetallic enhancement concept. Theoretical modeling was carried out by solving Fresnel equations for the multilayer stack of prism/Ag inner-metal layer/dielectric waveguide/Au outer-metal layer. The inner Ag layer couples incident light to a guided wave and makes more fields effectively concentrated on the outer Au surface. A substantial enhancement in resolution was experimentally verified for the model stack using a ZnS-SiO_2_ waveguide layer.

## 1. Introduction

Surface plasmons are charge density waves of free electrons which occur on a surface of a thin metal film interfacing with an adjacent dielectric and propagate along the interface. The amplitude of their enhanced electric field has a maximum at the metal surface and decays exponentially away from the interface. The surface plasmon is generally excited through a coupling of an evanescent field generated by a p-polarized light in attenuated total reflection (ATR) geometry [1,2]. At a certain incident angle where a phase matching condition is satisfied, *i.e.*, the component of the incident light’s wave vector parallel to the prism/metal interface equals that of the surface plasmon, the incident light gets transferred to a surface plasmon mode and creates a sharp dip in the reflectivity curve. Since the resonance condition in which surface plasmons are excited depends on a change in the surrounding environment adjacent to the thin metal film surface very sensitively, bio- and gas sensors using this technique have been extensively studied and developed [3–5].

Although a surface plasmon resonance (SPR) sensor finds use in many applications, there obviously exists a need to further enhance its sensing resolution to allow detection of low molecular weight molecules (a few hundreds of Daltons) and at trace level concentrations [6,7]. For early diagnosis of diseases and pathogen infection and rapid analysis of air and environmental pollution, development of such a high-resolution sensor is necessary. However, conventional SPR sensors which mainly use a Au single layer are known to show resolution limitations.

The resolution of a SPR sensor is improved as the linewidth of the SPR reflectivity dip curve decreases and a shift in the resonance angle or wavelength in response to the environmental index change increases [5,8]. With limited available metals for plasmonic application, most current efforts have been concentrated on decreasing the linewidth of the resonance curve [7–10]. Various configurations in a Kretschmann type device structure have been proposed and explored, mainly by designing the layer stack on hypotenuse face of a prism, which include long range SPRs (LRSPRs) [8,11], Au-Ag bimetallic film SPRs [10,12], waveguide coupled SPRs (WCSPRs) [7], *etc.* Particularly, the use of coupled mode of surface plasmon wave with other plasmonic or guided modes appears to provide most effective way of sharpening the SPR curve, thus enhancing the sensing resolution. Simple Au-Ag bimetallic SPRs cannot compete with the other configurations based on a coupled mode.

Besides designing the plasmonic stack mentioned above, many other system-wise approaches related to signal measurement and data processing were also suggested to improve resolution. Those include techniques based on phase modulation [13,14], double wavelength [15], wavelength division multiplexing [16], *etc*.

In this study, we present and analyze a plasmonic stack for the resolution enhancement of a SPR sensor based on a waveguide coupled configuration through combining a bimetallic approach. Theoretical modeling has been carried out by solving the Fresnel equations for a multilayer stack of metal/dielectric waveguide/metal geometry. A Ag inner-metal layer was expected to provide better performance as a low index coupling layer which couples and confines more of the light field into the dielectric waveguide, and then to an Au outer-layer, while minimizing optical loss related with the materials itself and field confinement. The SPR properties such as the resonance curve shape, local field near the Au surface, and the response to an environmental change were investigated and compared with those of a conventional SPR with a Au singlelayer and a WCSPR without Ag enhancement. Limit of detection (LOD) tests were also carried out with dilute sucrose solutions and these demonstrated a substantial gain in resolution.

## 2. Experimental

**Figure 1.**
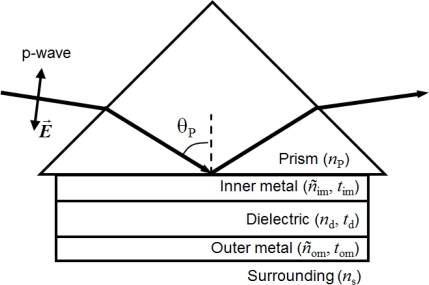
Schematic geometry of the present bimetallic WCSPR sensor used for theoretical calculation.

Figure 1 illustrates the model geometry of the bimetallic WCSPR (Bi-WCSPR) presented here. The model structures consist of a high index prism (SF10 with refractive index *n*_p_ = 1.723), Ag inner-metal layer, dielectric waveguide, and Au outer-metal layer. The presence of a Au outer surface maintains its merits of biocompatibility and material durability. Theoretical simulations of SPR reflectivity were carried out by solving the Fresnel equations for the multilayer stack and other conventional configurations with commercial optical thin film software (SCI Film Wizard^TM^) [17]. Several combinations of Ag to Au ratio as well as the thicknesses of each constituent layers were optimized in a water buffer (*n*_s_ = 1.332) for a p-polarized incident light of 632.8 nm to have sharp resonance curves and minimum reflectivity dips. As a waveguide, we adopted a high index ZnS-SiO_2_ (*n*_d_ = 2.198) due to its desirable properties of providing good adhesion to noble metals and a smooth interface between them. Besides, its high refractive index enables the total stack to be thin enough for the practical embodiment.

The optimized stacks were fabricated on the hypotenuse plane of a SF10 prism at room temperature by the RF magnetron sputtering method. The working pressure was 0.67 Pa (5 mTorr) in a pure Ar atmosphere. The RF power was set to 80 W for the ceramic ZnS-SiO_2_ target and 20 W for metallic Ag and Au. All targets are 2 in. in diameter.

The SPR reflectivity curves and the response to an environmental change were analyzed using a home-made system setup. The measurement was performed on a high precision 2-axis rotation stage (Kohzu). A monochromatic He-Ne laser of 632.8 nm wavelength was linearly polarized to give p-polarization and incident onto the hypotenuse plane of SF10 prism where the plasmonic stacks were pre-deposited. Reflected intensity was then monitored using a Si photodetector and a lock-in-amplifier (EG&G Instruments model 7265). Sensitivity and LOD tests were carried out using sucrose solutions prepared on a weight/weight percentage basis by dissolving d-(+)-sucrose (Sigma Aldrich) in de-ionized water at different concentrations. The refractive indices of the sucrose solutions were calibrated at room temperature by an Abbe-type refractometry [18]. A flow cell with a chamber volume of 18 μL was attached to the sensor surfaces and the sucrose solutions were delivered to the flow cell with a flow rate of 20 μL/min using a syringe pump (PHD2000, Harvard Apparatus, Holliston MA, USA).

## 3. Results and Discussion

**Figure 2.**
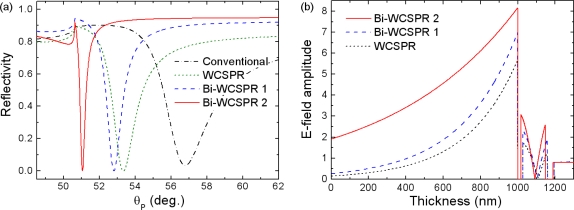
**(a)** Simulated SPR reflectivity curves and **(b)** local field distribution near the Au surface for some model configurations, where the WCSPR, Bi-WCSPR1, and Bi-WCSPR2 represent the multilayer stacks of SF10 prism/Au(35 nm)/ZnS-SiO_2_(131 nm)/Au(28 nm) (dotted line), SF10 prism/Ag(35 nm)/ZnS-SiO_2_(131 nm)/Au(28 nm) (dashed line), and SF10 prism/Ag(42 nm)/ZnS-SiO_2_(131 nm)/Au(16 nm) (solid line), respectively. The conventional SPR with a single Au layer (50 nm) was also included for comparison (dot-dashed line).

Figure 2(a) shows the SPR reflectivity curves calculated as a function of incident angle, θ_P_, in the incident medium of the SF10 prism for several model stacks. The optimum thickness of ZnS-SiO_2_ waveguide was determined to be 131 nm for all three WCSPR configurations. The complex refractive indices of Au and Ag used are 0.2184 + 3.5113*i* and 0.082 + 4.1563*i*, respectively. The reflectivity curve labeled as WCSPR represents the case where both the inner- and outer-metal layers are composed of Au and have respective thicknesses of 35 and 28 nm. Bi-WCSPR1 configuration has the multilayer stack of SF10 prism/Ag(35 nm)/ZnS-SiO_2_/Au(28 nm)/water buffer, the same as that of the WCSPR, except that the inner Au layer was replaced by Ag. Bi-WCSPR2 represents the case of increased ratio of Ag to Au, *i.e.,* 42 nm to 16 nm. A conventional SPR using a single Au layer of 50 nm thickness was also included for comparison.

As observed in Figure 2(a), the line width of the reflectivity curves is broadest in the conventional SPR and becomes narrower in the configurations using a coupled mode. The sharpness of the reflectivity curve is more significantly enhanced when the bimetallic approach is applied and the relative thickness ratio of Ag to Au increases. In addition, the resonance dip is shifted toward lower angles of incidence with increasing Ag/Au ratio, which guarantees a wider dynamic range (effective range of measurable refractive index). The calculation shown in Figure 2(b) also revealed the enhanced local electric field near Au surface implying the inner Ag promotes more field of incident light coupled concentrated on the outer Au surface.

**Figure 3.**
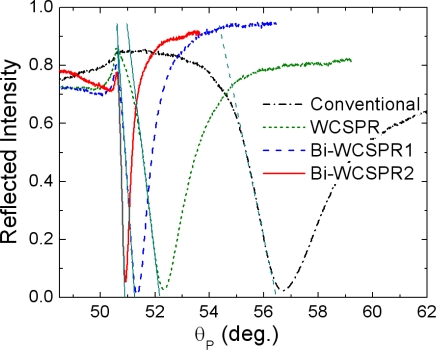
Measured SPR angular reflectivity curves for the model configurations fabricated with the geometrical parameters given in Figure 2(a).

Figure 3 shows the experimental reflectivity curves measured for the SPR configurations fabricated on the SF10 prism base using the geometrical parameters described above. The sequential stacks of the inner Ag (or Au) layer, the ZnS-SiO_2_ dielectric waveguide, and the outer Au layer are deposited at room temperature by RF magnetron sputtering. The overall profiles seem to be in good accordance with the simulation results. Some discrepancies observed might be ascribed to the uncertainty in refractive indices of thin metal layers and the thickness variation of constituent layers.

As confirmed in Figure 3, it is clear that the use of inner Ag layer instead of Au further enhances the sharpness of the WCSPR. Especially, the reflectivity curve of Bi-WCSPR2 is 7.68 times steeper (dR/dθ_P_ = 3.4266/°) when evaluated by the slope of linear portion of dip curve at low angle side and 9.62 times narrower in full width at half maximum (FWHM = 0.3483°) than those of conventional SPR (dR/dθ_P_ = 0.4463/°, FWHM = 3.3508°). Since the operations of a SPR sensor are performed by measuring a change in the reflectance dip curve in response to an environmental refractive index change, the sensing resolution, *i.e.*, the lowest refractive index change detectable is expected to be greatly enhanced due to the drastic reduction in the curve width.

To demonstrate the enhanced resolution, we analyzed the response of the present sensor configurations to the varying concentrations of sucrose solution with calibrated refractive indices. The sucrose solutions were prepared on a weight/weight percentage basis by dissolving 0, 1, 2, 3, 4, and 5 wt% d-(+)-sucrose in de-ionized water. The corresponding refractive indices determined at room temperature by an Abbe-type refractometry [18] were 1.3308, 1.3324, 1.3339, 1.3354, 1.3370, and 1.3389, respectively. A flow cell was attached to the sensor surfaces and the sucrose solutions were delivered to the flow cell using a syringe pump.

**Figure 4.**
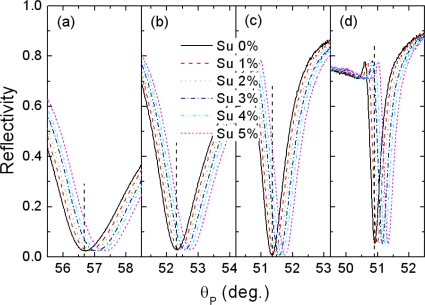
Measured SPR reflectivity curves with increasing the concentration of sucrose solution for each configuration shown in Figure 3, **(a)** the conventional SPR, **(b)** WCSPR, **(c)** Bi-WCSPR1, and **(d)** Bi-WCSPR2. The arrow lines indicate the position of reflectivity minima observed at pure water.

Figure 4 shows the SPR reflectivity curves measured at different sucrose solution concentrations for each configuration. The SPR angles, at which the reflectivity dip occurs, shift towards the higher incident angle with increasing refractive index of the sucrose solution, proportional to the concentration. Although the sensitivity defined as an angle shift with respect to the refractive index change, dθ_P_/dn is observed to be somewhat higher in the case of the conventional SPR (dθ_P_/dn = 76.34°) [Figure 4(a)] compared with that of the Bi-WCSPR2 (θ_P_/dn = 53.31°), it is clear that the enhanced sharpness of SPR dip in the coupled mode configurations would increase a signal-to-noise ratio and make a very slight angular shift to be resolved, especially when operated in an angular and/or intensity interrogation modes of detection and SPR imaging [19]. Since the resolution of a SPR sensor is closely linked to the marginal reflectance change resolved from the noise level, it can be estimated to be proportional to the value of (dθ_P_/dn) × (dR/dθ_P_). The higher magnitude of incremental change in the dip curve sharpness dR/dθ_P_ relative to that of sensitivity guarantees a gain in resolution. From this viewpoint, the resolution of Bi-WCSPR2 stack is expected to be 5.36 times higher than that of a conventional sensor. According to the literature reports [5,8], the resolution of a SPR sensor is correlated with both of sensitivity and width of SPR dip, and only weakly dependent on the choice of modulation.

**Figure 5.**
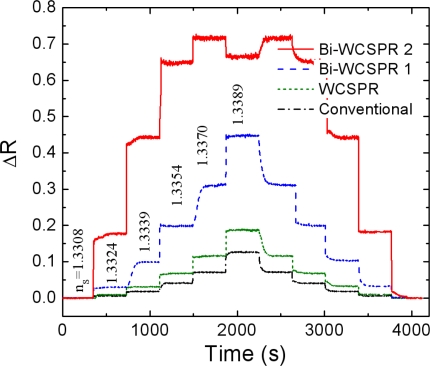
Temporal responses of the SPR reflectivity minima initially in pure water for each configuration as the sucrose solutions with different concentration were injected to the sensor surfaces. The refractive index of the water-sucrose solution was varied in steps from 1.3308 to 1.3389 and then back to 1.3308. The staircase profiles correspond, from top to bottom, to the responses of Bi-WCSPR2, Bi-WCSPR1, WCSPR, and conventional SPR, respectively.

Figure 5 verifies the enhanced response of the present Bi-WCSPR sensors. The temporal responses of the reflected intensity measured at the angle of initial resonance were monitored during a cycle of periodic injection of sucrose solution with a series of concentrations. As expected from Figure 4, the relative change in reflectivity of the Bi-WCSPR configurations is significantly increased, exhibiting much larger ΔR even for a small change in the refractive index of the surrounding medium. Furthermore, the Bi-WCSPR2 undergoes saturation to a total reflection even when the solution concentration exceeds 3 wt%, and exhibits a reflectance of the multilayer stack itself observed below a critical angle. Finally, the staircase responses recover to the initial state as the injected concentration returns back to the pure water.

**Figure 6.**
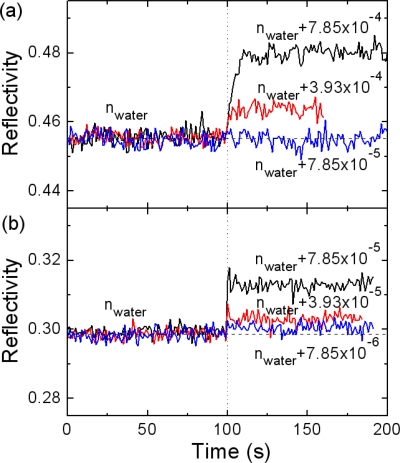
Temporal responses of the SPR reflectivity measured before and after injection of dilute sucrose solutions at the angle of highest slope of SPR dip curves for **(a)** conventional SPR and **(b)** Bi-WCSPR2 stack.

To demonstrate high resolution of the present Bi-WCSPR sensors, we carried out a LOD test as a way of determining the lowest refractive index of dilute sucrose solutions we can resolve from the water base. For this purpose, the temporal responses of the reflected intensity to the injection of dilute sucrose solutions were measured at the angle of highest slope of SPR dip for the Bi-WCSPR2 stack [Figure 6(b)] and compared with that of conventional SPR [Figure 6(a)].

Figure 6 shows the results monitored near the resolution limit of sucrose concentrations for each configuration. It is clearly seen that the refractive index change Δn of 7.85 × 10^−5^ undetectable with the conventional SPR is distinguished from the water buffer with the Bi-WCSPR2. Even an order of magnitude smaller index change of 7.85 × 10^−6^ appears to be resolved. This demonstrates a substantial gain in resolution for the Bi-WCSPR2 stack, almost an order of magnitude higher than that of conventional SPR. It should be mentioned here that the observed resolution verifies a significant enhancement relative to that of conventional SPR sensor and thus the resolution in absolute value should be further improved easily by tuning the stack design and upgrading the current measurement setup to a low noise system.

## 4. Conclusions

In summary, a novel bimetallic approach to enhance the resolution of WCSPR sensor was theoretically and experimentally investigated. The practical sensor with a slim stack was successively fabricated by adopting high index ZnS-SiO_2_ dielectric waveguide with good adhesion properties to noble metals. The use of a Ag inner coupling layer between the prism and the dielectric waveguide film was confirmed to have advantages in enhancing the sharpness of SPR curve as well as the local field at the Au surface interacting with analytes, thus improving the sensing capability, especially in terms of sensing resolution and dynamic range. As the relative ratio of Ag to Au increases, the linewidth of the reflectivity curve decreased more significantly, and the corresponding resolution of the sensor was verified to be greatly improved and able to detect smaller changes in the refractive index of the surrounding medium.
